# Epistemic anxiety and epistemic risk

**DOI:** 10.1007/s11229-022-03788-7

**Published:** 2022-07-29

**Authors:** Lilith Newton

**Affiliations:** School of Philosophy, Psychology and Language Sciences, University of Edinburgh, 3 Charles Street, EH8 9AD Edinburgh, UK

## Abstract

In this paper, I provide an account of epistemic anxiety as an emotional response to epistemic risk: the risk of believing in error. The motivation for this account is threefold. First, it makes epistemic anxiety a species of anxiety, thus rendering psychologically respectable a notion that has heretofore been taken seriously only by epistemologists. Second, it illuminates the relationship between anxiety and risk. It is standard in psychology to conceive of anxiety as a response to risk, but psychologists – very reasonably – have little to say about risk itself, as opposed to risk judgement. In this paper, I specify what risk must be like to be the kind of thing to which anxiety can be a response. Third, my account improves on extant accounts of epistemic anxiety in the literature. It is more fleshed out than Jennifer Nagel’s (2010a), which is largely agnostic about the nature of epistemic anxiety, focusing instead on what work it does in our epistemic lives. In offering an account of epistemic anxiety as an emotion, my account explains how it is able to do the epistemological work to which Nagel puts it. My account is also more plausible than Juliette Vazard’s (2018, 2021), on which epistemic anxiety is an emotional response to potential threat to one’s practical interests. Vazard’s account cannot distinguish epistemic anxiety from anxiety in general, and also fails to capture all instances of what we want to call epistemic anxiety. My account does better on both counts.

## Introduction

Epistemic anxiety is a phenomenon that has been posited to undermine the motivation for stakes-sensitive theories of knowledge, according to which what a subject knows is partly determined by what is at stake for her in the context.^1^ Jennifer Nagel argues that knowledge is not sensitive to stakes, rather our reluctance to attribute knowledge in high-stakes contexts is due to our expectation that subjects will think adaptively: they will invest greater cognitive resources into forming beliefs when error would be particularly costly, or true belief particularly beneficial. Nagel posits epistemic anxiety as a “force” (2010a, p. 408) that triggers subjects to gather information and reason more carefully in high-stakes contexts. However she does not have much to say about the nature of epistemic anxiety: what it is, and how it serves its function. My aim in this paper is to provide such an account of epistemic anxiety. I argue that epistemic anxiety is an emotional response to epistemic risk: the risk of believing in error.

The motivation for my account is threefold. First, it makes psychologically respectable a notion that has heretofore been taken seriously only by epistemologists: epistemic anxiety is simply a kind of anxiety, something we all agree is a genuine phenomenon. Second, my account contributes to recent philosophical work on risk, by specifying to which philosophical kinds of risk anxiety can be a response. Anxiety – the broad emotion of which I argue epistemic anxiety is a species – is understood by psychologists to be an emotional response to risk. Three accounts of risk have gained prominence in the literature: the probabilistic account, on which the risk of a negative event is determined by the likelihood of its obtaining; Duncan Pritchard’s (2015) modal account, on which risk is determined by the closeness of worlds in which a negative event obtains; and Philip Ebert, Martin Smith and Ian Durbach’s (2020) normic account, on which risk is determined by the most normal worlds in which a negative event obtains. I argue that anxiety is triggered in the presence of normic or probabilistic risk, but not modal risk. Nevertheless, I show my account of epistemic anxiety to be valuable from the perspective of anti-risk epistemology, which involves modal epistemic risk. Finally, my account improves on extant accounts of epistemic anxiety in the literature. It has greater explanatory power than Nagel’s: where Nagel says only that epistemic anxiety is a force, I argue that it is an emotion, hence has a particular representational, affective and motivational profile that explains how epistemic anxiety can do the epistemological work to which Nagel puts it. My account is also more plausible than Juliette Vazard’s (2018, 2021) account, on which epistemic anxiety is an emotional response to threat to one’s practical interests. Vazard’s account is problematic in two ways: it cannot distinguish epistemic anxiety from anxiety in general, and it cannot capture all instances of epistemic anxiety. My account does better on both counts.

The paper proceeds as follows. In the next section, I present an account of anxiety as a response to risk, on which anxiety is triggered in the presence of normic and probabilistic risk without having either notion encoded in its representational content. In § 3, I apply this picture to the epistemic realm, arguing that epistemic anxiety is an emotional response to epistemic risk, and that it is triggered in the presence of normic or probabilistic epistemic risk, but not modal epistemic risk. I introduce an objection from anti-risk epistemology: modal epistemic risk is what undermines knowledge, so if epistemic anxiety doesn’t track modal epistemic risk, how can it be epistemically valuable? I respond to this by appeal to normative coincidence: one cannot aim to reduce normic risk without aiming to reduce modal risk, so insofar as epistemic anxiety motivates us to reduce normic epistemic risk, it motivates us to reduce modal epistemic risk. In § 4, I compare my account of epistemic anxiety to Nagel’s and Vazard’s. I end by suggesting that future research could be done exploring the relationships between epistemic anxiety, doubt and inquiry.

## Anxiety and risk

Psychologists conceive of anxiety as an emotional response to *threat* or *risk* (see Lader & Marks 1973, Butler and Mathews 1987, Öhman 1993, Barlow, 2001, Kemeny & Shestyuk, 2008). It is standard to distinguish between *trait* and *state* anxiety. ‘State anxiety’ refers to emotional episodes of anxiety. These are short-lived affective responses to specific stimuli. ‘Trait anxiety’ refers to an individual’s disposition to experience state anxiety. An anxious person, someone with high trait anxiety, will be disposed to experience state anxiety more often than other people, or in response to a greater variety of stimuli. It is state anxiety with which I am concerned in this paper. Henceforth when I mean to refer to state anxiety, I will just use ‘anxiety’.

Risk is commonly characterised in terms of potential unwanted events: one faces a risk where one faces the possibility of an unwanted event obtaining. Pritchard defines risk events as “potential unwanted events” (2015, p. 436); Adam Bricker writes that “a risk is an unwanted possible event” (2018, p. 201); and Sven Ove Hansson 2018, notes that risk has been variously defined as “an unwanted event that may or may not occur”, “the cause of an unwanted event that may or may not occur” and “the probability of an unwanted event that may not occur” (2018).^2^ This is problematic, because what people want often does not line up with what is good or valuable. A depressed person may want to die; then any event in which she lives constitutes a risk event for her, and many events in which she dies do not. Pritchard notes this problem, writing that “whether an event is unwanted will be a subjective matter; one might actively want the plane to crash, for example, because one is suicidal” (2015, p. 437). However, he “set[s] this complication to one side and take[s] it as given” that risk events are potential unwanted events (437). I do not wish to set this problem aside, so, taking a hint from the risk analysis literature, will use the terminology of *negative* events (see Jensen 2012, pp. 436-7). Negative events are events whose obtaining would be disvaluable in some way, but which need not be unwanted: they might be harms (Möller, 2012, p. 74), or events that involve loss of moral, aesthetic, or other kinds of value. I intend for ‘negative’ to be a placeholder for whatever way risk events are disvaluable. In what follows, I use ‘risk events’ to name potential negative events, and ‘risk-possibilities’ to name possibilities in which such events obtain.

Anxiety is an emotional response to risk. It functions to direct the experiencer’s attention towards some risk-possibility and motivate her to take steps to avoid or reduce the relevant risk. There are three elements to anxiety that enable it to function in this way. First, anxiety has representational content. It represents some event as possible, in a robust sense. That is, when one experiences anxiety about an event E, E is not represented to one as merely metaphysically possible: as something that happens in some possible world, however bizarre is that world, or however incompatible that world is with how one knows the actual world to be. Rather, E is represented to one as something that, for all one knows, might come to obtain in the actual world. We can say, then, that one’s anxiety represents E to one as *epistemically*, not merely metaphysically, possible: possible relative to what one (or perhaps some wider group of subjects) knows.^3^ Pritchard argues that the relevant sense of ‘possibility’ is even more restrictive than this: not only must a risk-possibility be epistemically possible, it must be “realistic”, where this means it is “something that could credibly occur” (2015, p. 439; see also Grimm 2015, p. 132). In what follows, I assume that anxiety represents an event to its experiencer as *at least* epistemically possible. One’s anxiety also represents that event to one as negative: disvaluable in some way. Thus anxiety represents an event to one as a potential negative event – that is, *as* a risk event. We can put this point by saying that the *formal object* of anxiety is risk: risk is what anxiety is about.

Emotions have intentionality, or aboutness, in two ways. First, they are about particular objects or states of affairs. If I feel affection towards my cat, my affection is *about* him. This sense of aboutness is that of having a material object. Second, emotions represent their material objects as (dis)valuable in particular ways. My affection towards my cat represents him as worthy of affection, as loveable. This sense of aboutness is that of having a formal object. While the material object of an emotion is different in different cases, the formal object of an emotion cannot vary between different token experiences of that emotion. This is because an emotion’s formal object determines just what emotion it is: emotions are (at least partly) individuated by their formal objects. In the case of anxiety, though the material object of anxiety can be any number of things, its formal object is always risk. For example, one can be anxious about missing one’s train, about responding to an email, about the supermarket being out of goat’s cheese. In all these cases, one’s anxiety represents the event in question to one as a risk event: a potential negative event. If one’s emotional experience did not represent a situation as involving risk, it would not be an experience of anxiety.

The second element of anxiety that enables its function is its unpleasant affective aspect. Anxiety is experienced as aversive: as “tension, unease and concern” (Vazard, 2018, p. 142). The representational and affective elements combine to give anxiety its third element: motivation to risk-reduction behaviours. To see the three elements of anxiety in action, consider an example. If I am anxious about catching SARS-CoV-2, the virus that causes COVID-19, my catching SARS-CoV-2 is represented to me as negative and as robustly possible in the way described above. My anxiety has an unpleasant affective aspect: when I think about situations in which I could catch SARS-CoV-2, I feel uneasy and tense. I thus experience my anxiety as aversive, and consequently am motivated to engage in risk-reduction behaviours: to avoid gathering with other people indoors; to wear a mask when I cannot avoid this; and so on. This motivation is immediate: I do not need to have any independent desire to reduce the relevant risk in order to be motivated to do so; rather, my anxiety provides the motivational power required for me to try to reduce the relevant risk.

From the picture taking shape, it should be clear that, perhaps contrary to folk thinking about anxiety, anxiety is a very valuable emotion in our emotional toolbox. Appropriately experienced anxiety brings to one’s attention possibilities whose obtaining would be negative, and immediately motivates one to take steps to guard against those possibilities’ obtaining. Consider Charlie Kurth’s (2018) discussion of the anxiety experienced by neurosurgeon Henry Marsh. Marsh “sees [his anxiety] as the manifestation of his accumulated surgical expertise: when determining whether to remove more of a tumor – at the risk of damaging healthy brain tissue – he is guided by his anxiety” (2018, p. 3). When he starts feeling anxious, he stops operating (see Marsh 2004). Marsh’s anxiety here focuses his attention on a risk event, that of damaging healthy brain tissue, and motivates him to avoid the risk by stopping the surgery. His anxiety is thus very valuable, given his goal of removing tumours without damaging healthy brain tissue.^4^

More generally, anxiety can be positively evaluated both in terms of its fittingness, and its utility. As the formal object of anxiety is risk, anxiety will be fitting when it is a response to genuine risk, and unfitting when there is no risk present. For example, Marsh’s anxiety is fitting: there is a genuine risk of damaging healthy brain tissue, and his anxiety is a response to that risk. In contrast, the anxiety experienced as part of generalised anxiety disorder (GAD) is unfitting: it is either undirected, in the case of “free-floating anxiety”, or it is directed at everyday events that do not involve risk (ICD-11, World Health Organisation 2018). The utility of anxiety is a matter of whether it helps or hinders one’s goals, and the extent to which it does this. Marsh’s anxiety is useful because it enables him to remove as much of a tumour as possible without damaging healthy brain tissue. Again, in contrast, the GAD sufferer’s anxiety is unhelpful as it causes impairment in social, educational, occupational or other areas of functioning (WHO 2018).

If anxiety is fitting only in response to genuine risk, we must get clearer on what exactly risk is. I have so far said that risk events are ‘potential negative events’: negative events that obtain in some epistemically possible world. Each philosophical account of risk can accept this claim. The three accounts diverge over what it is that determines the level of risk of some risk event. The “standard” philosophical account of risk – so called by Pritchard (2015, p. 436), Bricker (2018, p. 200), and Ebert et al., (2020, p. 432) – has it that the potentiality of risk events is a matter of probability: an event E is a risk event iff E is a negative event with a non-zero probability of obtaining. High-risk events have a higher probability of obtaining, low-risk events have a lower probability of obtaining, and there is a continuum of riskiness between these extremes. The relevant notion of probability here is *evidential* or *epistemic* probability: probability relative to a body of evidence (see Ebert et al., 2020, p. 433). Evidential probability is contrasted with *physical probability*, which can be thought of as the brute frequency with which tokens of some event-type obtain, not relative to any subject’s thinking nor to any body of evidence; and *subjective probability* or *credence*, which measures how strongly a subject believes a given proposition (Mellor, 2005, p. 8). Evidential probability is not wholly objective, like physical probability, because it is always relative to a body of evidence. But neither is it subjective, like credence. Evidential probability is objective to the extent that, given a body of evidence, there is a fact of the matter about what is the evidential probability of E’s obtaining. Then interpreting the probabilistic account in terms of evidential probability “reflects the fact that this is an account of risk as an objective phenomenon”, to use Pritchard’s (2015, p. 440) words, without making risk something wholly beyond our ken.

Pritchard (2015) and Ebert et al., (2020) challenge the standard probabilistic account of risk. They argue that risk is not determined solely by the probability of a negative event’s obtaining, but rather is (at least sometimes) a matter of how easily a negative event could obtain (Pritchard), or the extent to which that event’s obtaining would call out for explanation given a body of evidence (Ebert, Smith and Durbach). Pritchard calls his picture of risk the *modal* account, and Ebert, Smith and Durbach call theirs the *normic* account.

Pritchard motivates his account by appeal to a pair of cases in which it is stipulated that the probability of a negative event’s obtaining is equal in both, but in which the event could obtain more easily in one case than in the other. These are his bomb cases:

### Case 1

An evil scientist has rigged up a large bomb, which he has hidden in a populated area. If the bomb explodes, many people will die. There is no way of discovering the bomb before the time it is set to detonate. The bomb will only detonate, however, if a certain set of numbers comes up on the next national lottery draw. The odds of these numbers appearing is fourteen million to one.

### Case 2

[All is the same as in Case 1, but] The bomb will only detonate if a series of three unlikely events obtains. First, the weakest horse in the field at the Grand National, Lucky Loser, must win the race by at least ten furlongs. Second, the worst team remaining in the FA Cup draw, Accrington Stanley, must beat the best team remaining, Manchester United, by at least ten goals. And third, the queen of England must spontaneously choose to speak a complete sentence of Polish during her next public speech. The odds of this chain of events occurring are fourteen million to one. (2015, p. 441)

Pritchard argues that there is a much higher risk of the bomb going off in Case 1 than in Case 2, despite the identical odds, because in Case 1 the bomb blast “could very easily occur. All it would take for the bomb to go off … is for a few coloured lottery balls to fall in a certain configuration” (442). In Case 2, in contrast, the bomb blast couldn’t easily occur. For this to happen, not one, but three, incredibly far-fetched events must take place. The probabilistic account cannot explain why the risk of the bomb going off in Case 1 is, at least according to Pritchard’s intuitions, much higher than in Case 2, as it is stipulated that the probability of this event obtaining is the same in both cases.^5^ Pritchard concludes that the probabilistic account is “highly problematic”, as it cannot capture “our natural judgements … about risk” (447).

Pritchard takes himself to have shown that the probabilistic account is “fundamentally misguided” (436). In its place, he argues we should endorse his modal account of risk, on which the risk of a negative event E is determined by the closest worlds in which E obtains. A world is close to the actual world if it is similar to the actual world, and worlds become more distant to actuality as they become more dissimilar, with similarity being a matter of how much needs to change to get from the actual world to a given possible world. On Pritchard’s modal account of risk, the closer is the closest world(s) in which a negative event E obtains, the riskier is E (447). E is high-risk if, keeping relevant initial conditions fixed,^6^ E obtains in a close possible world (2016, p. 562). As the closest world in which E obtains becomes more distant, the riskiness of E lessens, until eventually E is so remote as to not constitute a risk event.

Ebert, Smith and Durbach agree with Pritchard that there is more to risk than the probabilistic account has it. However they disagree with Pritchard in two respects. First, they don’t accept that Pritchard has shown the probabilistic account to be fundamentally misguided. They argue that there are cases for which the probabilistic account can deliver the intuitively correct verdict, but the modal account cannot. As example, they offer a new bomb case:

An evil scientist has rigged up a large bomb, which he has hidden in a populated area. If the bomb explodes, many people will die. There is no way of discovering the bomb before the time it is set to detonate. The bomb will only detonate, however, if six specific numbers between 1 and 99 come up on the next national lottery draw. The odds of these six numbers appearing are roughly one billion to one. (2020, p. 446)

Call this ‘Case 3’. The odds of the bomb going off in Case 3 are much lower than in Case 1: one in one billion, compared to one in 14 million. The probabilistic account judges that the risk of the bomb going off is much higher in Case 1 than in Case 3. However, the closest world in which the bomb goes off in Case 3 is equally close as in Case 1: just as in Case 1, all that is required for the bomb to go off in Case 3 is that a few coloured balls fall in a particular configuration. Thus the modal account judges that the risk of the bomb going off is equal in Cases 1 and 3.

This is at least as hard to swallow as the probabilistic account’s verdict that the risk of the bomb blast is equal between Cases 1 and 2. This suggests to Ebert, Smith and Durbach that the probabilistic notion of risk still has an important place in our dealings with risk. They therefore recommend that we endorse pluralism about risk, according to which different philosophical accounts of risk are understood as different, but equally legitimate, precisifications of our intuitive notion of risk (2020, p. 449). However they argue that Pritchard’s modal account is problematic in other ways, and that their own normic account can capture the benefits of Pritchard’s account without these untoward elements.^7^ So their risk pluralism does not involve endorsing the modal account. This is their second point of disagreement with Pritchard.

On Ebert, Smith and Durbach’s normic account, the risk of a negative event E isn’t determined by the closeness of worlds in which E obtains, but by the *normalcy* of those worlds. The notion of normalcy appealed to is that developed by Smith (2016) in terms of calling out for explanation. Normal conditions don’t call out for any special explanation, relative to a body of evidence, while abnormal conditions do. For example, if I say “Iain would normally be home by six,” part of what I mean by this is that, if Iain failed to be home by six, some explanation would be required (2016, p. 39). Perhaps his car broke down, perhaps there was traffic, perhaps he stopped for ice cream. In any case, if Iain is normally home by six, his failing to be home by six calls out for explanation. Possible worlds can be ordered by their normalcy. The most normal worlds are those whose obtaining would call out for the least explanation, given a body of evidence. Worlds become more abnormal as their obtaining calls out for more explanation. On the normic account of risk, the risk of an event E is determined by the most normal worlds in which E obtains. The more normal are these worlds, the higher the risk of E (2020, p. 444).

The normic account of risk issues the same verdicts as the modal account for the bomb cases. Case 1 is judged to be riskier than Case 2, as it would not be abnormal for the detonation-triggering numbers to come up in the lottery, whereas it would be very abnormal for even one, let alone all three, of the triggering conditions to obtain in Case 2. Then the most normal worlds in which the bomb goes off in Case 1 are very normal, whereas the most normal worlds in which the bomb goes off in Case 2 are very abnormal. So the risk of the bomb going off is much higher in Case 1 than in Case 2. Case 1 and Case 3 are again judged to be equally risky: just as the bomb blast worlds are (presumed to be^8^) equally close in Case 1 and Case 3, these worlds are equally normal. Given our evidence, the bomb blast in Case 3 would require no more explanation than it would in Case 1: in both cases, the detonation-triggering numbers might as well come up as any other series of numbers. So there is still a place for the probabilistic account of risk in Ebert, Smith and Durbach’s risk pluralism.

An important difference between the normic and the probabilistic accounts of risk, on the one hand, and the modal account on the other, is that only the former two have it that risk is always relative to a body of evidence. This difference can be thought of in terms of objectivity. The modal account of risk is fully objective, in that it has it that risk is a brute fact about the world, and is neither relative to any subject’s beliefs or feelings about risk, nor to any body of evidence. Though a body of evidence might suggest that some worlds are close and others further away, which worlds are close is not determined by evidence. The normic and probabilistic accounts deliver notions of risk that are less than fully objective, though not subjective either. There is a fact of the matter about what is the normic or the probabilistic risk of an event E’s obtaining, but only relative to a body of evidence. In contrast, there is a fact of the matter about what is the modal risk of E’s obtaining, and this is not relative to any body of evidence (except, perhaps, all the evidence in the world).^9^

## Tracking risk

If anxiety is an emotional response to risk, does that mean that anxiety tracks risk of all different kinds? I argue that it does not. In this section, I argue that anxiety can track normic and probabilistic risk, but because modal risk is fully objective – it is neither relative to any subject’s beliefs or feelings about risk, nor to any body of evidence – it is not something that anxiety can track. This might surprise Pritchard, who appeals to the affective situation of the subjects in his bomb cases to support the verdicts issued by his modal account. Regarding Case 1, in which the bomb blast would be triggered by lottery, Pritchard writes that “[nobody] who knew about the bomb plot would be sitting comfortably while watching the next lottery draw” (2015, p. 442). However in Case 2, there would be no “corresponding cause for alarm” (441). These affective responses can be understood in terms of anxiety: the subjects in Case 1 are anxious about the bomb going off, while the subjects in Case 2 are not. This seems to suggest that anxiety can track modal risk. But note that in these cases, normic risk and modal risk correspond: in Case 1, both normic and modal risk are high, while in Case 2, both are low. What we need to see whether anxiety tracks modal risk are cases where modal and normic risk diverge: where modal risk is high and normic risk low, or vice versa. I will now offer two such cases, and argue that in these cases, anxiety tracks normic and not modal risk.

Recall Pritchard’s Case 2, in which the bomb will go off only if (a) Lucky Loser wins the Grand National by at least ten furlongs; (b) Accrington Stanley beats Manchester United by at least ten goals; (c) the queen of England spontaneously chooses to speak a complete Polish sentence in her next public speech. Suppose that I am in a case like Case 2, but in which, unbeknownst to me, Lucky Loser has been given performance enhancing drugs, while all the other horses have been given tranquilisers; all of Manchester United’s defenders have broken their toes; and the queen of England has been watching lots of Polish films and keeps bursting out with quotes from her favourites at inopportune moments. In this new case – call it ‘Case 4’ – although the normic risk of the bomb going off, relative to my evidence, is the same as it was in Case 2, the modal risk is much higher. But this would make no difference to the level of anxiety I feel about the bomb blast. I would remain as sanguine about the bomb blast in Case 4 as I would be in Case 2. Thus in Case 4, in which normic risk is low and modal risk is higher, anxiety seems responsive to normic and not modal risk.

Now imagine a case like Case 2, but in which I have received misleading information from an ordinarily trustworthy friend that things are as described in Case 4: that is, she has told me that Lucky Loser has been given performance enhancing drugs, that Manchester United’s defenders have all broken their toes, and so on. However none of what she tells me is true.^10^ In this case, given my evidence (which now includes that an ordinarily reliable friend has told me that Lucky Loser has been given performance drugs, and so on), the normic risk of the bomb going off is higher than in Case 2. The modal risk, however, is the same: the same amount would have to change in this case – call it ‘Case 5’ – to get from the actual world to the bomb blast world as in Case 2. But in Case 5, I would be more anxious about the bomb blast than I would in Case 2. Again, my anxiety seems responsive to normic and not modal risk.

Because the modal account of risk has it that risk is determined by what goes on in close possible worlds, and subjects can lack evidence, or indeed have misleading evidence, about what goes on in those worlds, the modal account makes it the case that what determines risk is not something that is entirely epistemically accessible to subjects. Our epistemic access to closeness orderings on worlds is incomplete. As we have just seen, sometimes our evidence will suggest that a world is close, when it is in fact distant; and sometimes our evidence suggests that a world is distant, when it is in fact close. Where a risk-possibility is close but one’s evidence does not suggest this, one doesn’t experience anxiety; and where a risk-possibility is distant but one’s evidence suggests that it is close, one does experience anxiety. Then anxiety doesn’t track modal risk. The kinds of risk to which anxiety can be a response cannot be fully objective in the way that the modal account has it. Rather, the risk that anxiety tracks must be evidence-relative.

This doesn’t mean that the experience of anxiety must be mediated by conscious reflection on one’s evidence. I think what is more plausible is that, in the cases just discussed, anxiety is triggered by specific stimuli in one’s environment to which one has epistemic access, and facts about these stimuli also feature in one’s body of evidence, which determines normic risk. Then when one’s situation involves high normic risk, one also feels anxious. We could say that anxiety tracks high normic risk in that both are common effects of a single cause: some feature(s) of one’s environment to which one has epistemic access both triggers anxiety and leads to certain facts being in one’s body of evidence, which determines that the normic risk is high. In some cases, however, I do think anxiety will be generated by conscious reflection on one’s evidence; for example, when probabilistic risk is high. A (non-pathological) subject will feel anxious when she plays a death lottery of the kind described in Shirley Jackson’s (1991) *The Lottery*, because this situation is normically very risky. But she would feel more anxious if she knew there were only 100 participants in the lottery than if she knew there were 100 million participants. In this case, her reflection on her evidence alerts her to the higher probabilistic risk of her death in the case of the smaller lottery, and her heightened anxiety is a response to this greater risk.

Psychologists working on risk have suggested that two different systems are involved in our risk judgements: an affective system and a cognitive system (Loewenstein et al., 2001; Shulman & Cauffman, 2014). Ebert, Smith and Durbach suggest that their risk pluralism could be merged with this ‘dual system’ approach to risk judgement, generating a picture on which normic risk is tracked by the affective system, and probabilistic risk by the cognitive system (2020, p. 449). If I am right, however, this is too quick. For anxiety is responsive to both normic and probabilistic risk, just in different ways: in the face of significant probabilistic risk, anxiety can be generated by reflection on one’s evidence; whereas in the face of high normic risk, anxiety is triggered directly by features of one’s environment. Then the affective system may still be involved in a subject’s making a probabilistic risk judgement; that a subject’s risk judgement has been informed by emotional experience does not mean that the kind of risk present in the subject’s environment is normic, rather than probabilistic.

Note that claiming that anxiety tracks high normic and probabilistic risk, but not modal risk, is not to claim that anxiety represents a situation as involving risk of the former two kinds. On my picture, anxiety simply represents a situation as involving risk: one’s situation is represented to one as implying the robust possibility of a negative event obtaining. This feels affectively unpleasant, and one is thereby motivated to try to take steps to guard against this event’s obtaining. But it is not part of the representational content of one’s emotional experience that the event’s obtaining would be normal, or probable. What kind of risk is present when anxiety is generated need not be part of the content of one’s emotional experience for anxiety to function in the way that it does.

## Epistemic anxiety and epistemic risk

In this section, I apply this picture of anxiety and risk to the epistemic realm, arguing that epistemic anxiety is an emotional response to epistemic risk: the risk of believing in error. Like anxiety in general, epistemic anxiety tracks normic and probabilistic (epistemic) risk, not modal risk. I raise an objection to my account from anti-risk epistemology: reducing modal epistemic risk is what matters for knowledge, so if epistemic anxiety doesn’t track modal epistemic risk, how can it be epistemically valuable? My answer is that epistemic anxiety is valuable because the goals of reducing normic epistemic risk, on the one hand, and reducing modal epistemic risk, on the other, are *normatively coincident*: one cannot aim for one without aiming for the other. Then insofar as epistemic anxiety motivates one to reduce normic epistemic risk, it motivates one to reduce modal epistemic risk, so is valuable from the perspective of anti-risk epistemology.

Epistemic risk is standardly conceived as the risk of believing in error (Collins, 1996, p. 208; Wright 2004; Lasonen-Aarnio, 2008; Smith, 2012; Pritchard, 2016; Babic, 2019; Vazard, 2021, p. 6921). Epistemic risk can be understood as a subspecies of risk, where the relevant risk event is one of forming or holding a false belief. Like risk in general, the broad notion of epistemic risk can be precisified along probabilistic, modal or normic lines. One’s forming or holding a belief is probabilistically epistemically risky if it is likely, given one’s evidence, that the belief one would thereby come to have would be false;^11^ it is modally epistemically risky if, holding fixed initial conditions, there are close worlds where the belief is false; and it is normically epistemically risky if there are normal worlds, relative to one’s evidence, in which the belief is false.

I propose that epistemic anxiety is to epistemic risk as anxiety in general is to risk. That is, epistemic anxiety is an emotional response to epistemic risk: it represents one’s cognitive situation as involving epistemic risk; it is affectively unpleasant; and it motivates subjects to behaviours aimed at reducing the relevant epistemic risk. The formal object of epistemic anxiety is epistemic risk. The material object is some particular epistemic risk event: some instance of forming or holding a false belief. For example, suppose that I hold a ticket in a fair, ten million-ticket lottery. I consider the odds that my ticket is a loser, and reason that this is so overwhelmingly likely that it would be rational for me to believe it. However, a niggling unease prevents me from forming the belief that my ticket is a loser: after all, one ticket will win, and it might as well be mine as any other. Here, I suggest that I am experiencing epistemic anxiety. The event of my forming a false belief (because I believe my ticket is a loser when it is, in fact, the winner) is *represented* to me as an epistemic risk event; I have an *aversive affective experience* (niggling unease); and these representational and affective aspects of my emotional experience combine to *motivate* me to take steps to reduce epistemic risk, by suspending judgment on whether my ticket is a loser. In this case, the formal object of my emotional experience is epistemic risk, and its material object is the event of forming a false belief that my ticket is a loser.

Like anxiety in general, epistemic anxiety will track normic and probabilistic epistemic risk, though its representational content won’t be so precise: when one experiences epistemic anxiety, one’s cognitive situation is simply represented to one as epistemically risky. In the lottery example just discussed, the probabilistic risk is very low, but the normic (and indeed the modal) risk is high. The emotional experience I have that prevents me from forming the belief that my lottery ticket is a loser does not have as part of its representational content that the normic or modal risk of forming this belief is high. It simply represents forming the belief to me as epistemically risky: as a potential, negative epistemic event.

Again, like anxiety more generally, epistemic anxiety is not responsive to modal epistemic risk. If I receive a ticket for an upcoming ten million-ticket lottery, and my ordinarily trustworthy friend tells me that it is a ticket for last week’s lottery and that it lost, then I will believe that this ticket is a loser. This belief is modally very risky. Given that this ticket is not, in fact, a ticket for last week’s lottery, but for an upcoming lottery, my belief could very easily be false (because this ticket could easily be the winner). However, I don’t experience epistemic anxiety regarding this belief. This is because it is not normically or probabilistically risky, given my evidence, which includes what my ordinarily trustworthy friend has told me.

Here, a worry arises. If epistemic anxiety is not responsive to modal epistemic risk, then is it redundant from the perspective of anti-risk epistemology? It is widely held amongst contemporary epistemologists that knowledge is incompatible with significant epistemic risk. This is the central thesis of Pritchard’s (2016) anti-risk epistemology. Further, safety theorists in general can be understood as anti-risk epistemologists, since they hold that a belief that could easily have been false, given how it was formed, cannot constitute knowledge (Sainsbury, 1997, p. 913; Sosa 1999; Williamson, 2000). But it is crucial that anti-risk epistemology be formulated in terms of *modal* epistemic risk. Consider again the example from the previous paragraph. My belief that my ticket is a loser (because my friend told me it is a ticket for last week’s lottery) cannot constitute knowledge: it too easily could be false. That is, it is too epistemically risky. But as noted, it is neither normically nor probabilistically epistemically risky. It is only modally epistemically risky. It is modal epistemic risk that is knowledge-undermining. But if epistemic anxiety does not track modal epistemic risk, as I argue, then it seems that it cannot help us to reduce the kind of epistemic risk that matters when it comes to having knowledge. From the perspective of anti-risk epistemology, then, epistemic anxiety is redundant.

However I argue that epistemic anxiety is not redundant from the perspective of anti-risk epistemology. Rather, experiencing epistemic anxiety is valuable, given our concern with achieving knowledge, because reducing normic epistemic risk and reducing modal epistemic risk are *normatively coincident* goals. The terminology of ‘normative coincidence’ is originally due to Crispin Wright (1992, p. 18). Two goals are normatively coincident if one cannot aim for one without aiming for the other. This doesn’t mean that one cannot achieve one goal without achieving the other, but that one cannot aim to bring about a situation in which one achieves one and not the other. Smith (2016, p. 9) gives an example to illuminate the notion. Suppose I am a member of a running club, and hold some of the best times in the club, but not the best. I am due to run a race. Two goals I might have for the race are (1) to set a new club record, and (2) to set a new personal best. Smith argues that (1) and (2) are normatively coincident, because the things I need to do to try to achieve (1) – keep fit, eat healthily, train hard – are just the things I need to do to try to achieve (2). I could end up achieving (2) without achieving (1). But I couldn’t aim to achieve (2) without aiming to achieve (1), and vice versa.

Smith argues that aiming for knowledge and aiming for justified belief are likewise normatively coincident goals (2016, p. 11). The things one must do to aim for knowledge – believe based on good evidence, suspend judgement in the face of defeaters, and so on – are exactly the kinds of things one must do to aim for (*ultima facie*) justification. One might end up achieving justified belief without achieving knowledge – this is how the predicament of the Gettiered subject is standardly understood. But this will be due to factors beyond one’s control. One cannot aim to bring about a situation in which one has a justified belief that P, but doesn’t know that P.^12^

Similarly, I argue that the goals of reducing normic epistemic risk and of reducing modal epistemic risk are normatively coincident. Which worlds one takes to be close will depend on how one’s evidence suggests the actual world to be. The worlds that one’s evidence suggests are close are those worlds in which there are few differences to how one’s evidence suggests the actual world to be. The steps one would take to try to eliminate close error-possibilities, so to reduce modal epistemic risk, would thus be to gather evidence about what the actual world is like; to reason about what are the ways that P could most easily be false, given this evidence – that is, to imagine worlds in which P is false, but there are as few other differences to how one takes the actual world to be as possible; to gather evidence that rules out these error-possibilities; and to suspend belief when some of these error-possibilities remain compatible with one’s evidence. But these are exactly the steps that one would take to try to eliminate normal error-possibilities, so to reduce normic epistemic risk. Normal worlds are worlds whose obtaining calls out for the least explanation, given a body of evidence. Generally, the obtaining of worlds in which more things are different to the most normal worlds (the worlds whose obtaining would call out for no explanation) would require more explanation than worlds in which fewer things are different. Then a subject who is trying to eliminate normal worlds would have a body of evidence that includes propositions about how she takes the actual world to be; she would then reason about which error-possibilities could “just so happen” to obtain, given this body of evidence (Smith, 2016, p. 39) – that is, whose obtaining would call out for the least explanation; she would gather evidence that rules out these error-possibilities; and she would suspend judgement while some of these error-possibilities remained compatible with her evidence.

Which possibilities one’s evidence suggests are close will roughly align with which possibilities would not call for special explanation on one’s evidence. Abnormal events sometimes obtain in the actual world, so close worlds are not identical to normal worlds. However, where it is part of one’s evidence that an event that was abnormal – whose obtaining would have called out for explanation given one’s prior evidence – has occurred, worlds in which that event obtains are now more normal than worlds in which it does not. Given that one’s body of evidence now includes *that X event obtained* – call this proposition ‘P’ – the situation in which one’s body of evidence E, which includes P, is true but P is false would call out for more explanation than the situation in which E is true and P is true, because only the former requires that a contradiction be true, which is impossible. So even though abnormal events obtain in the actual world, when it is part of one’s evidence that such an event has obtained, worlds in which the event obtains are, in the end, (relatively) normal worlds, on one’s evidence. Thus I hold that trying to rule out normal worlds will be the same activity as trying to rule out worlds which one’s evidence suggests are close. Then to try to reduce normic epistemic risk, as epistemic anxiety motivates us to do, is to try to reduce modal epistemic risk, as anti-risk epistemology demands.

Again, this doesn’t mean that one cannot achieve one goal without achieving the other. One might succeed in reducing the normic epistemic risk of a belief by gathering evidence that is incompatible with all the most normal ways one’s belief would be false. But if some abnormal way in which one’s belief would be false is nevertheless actual, or very close to it, then one will have failed to rule out close error-possibilities and thus failed to reduce the modal epistemic risk of one’s belief (at least to the same extent as one has reduced normic epistemic risk). Suppose I believe that Mr Bond is not on the plane because I know that he missed its take-off, and I know that the plane hasn’t made any stops since then at which he could have got on. I have thus ruled out the most normal ways my belief could be false, and consequently it is not normically epistemically risky. But suppose that Mr Bond is, in fact, on the plane – he parachuted onto it, then climbed inside through the luggage hatch, shortly after take-off. Then I didn’t manage to reduce the modal epistemic risk of my belief. I didn’t rule out the closest way in which it might have been false – the way that actually obtains, so is maximally close.

In this case, and in many others, ruling out normal error-possibilities does not guarantee knowledge. Nevertheless, if I am right that the goal of reducing normic epistemic risk and the goal of reducing modal epistemic risk are normatively coincident, then in many situations in which you succeed in ruling out all the most normal error-possibilities, you will also rule out all the closest, so be able to know by the lights of anti-risk epistemology. If you have ruled out the most normal possibilities, and the world is obliging, then you will have ruled out the closest possibilities, too. Thus experiencing epistemic anxiety is valuable from the perspective of anti-risk epistemology, even though epistemic anxiety doesn’t track modal epistemic risk. Epistemic anxiety alerts subjects to normal error-possibilities, and motivates them to take steps to take steps to eliminate these possibilities. Once you have eliminated these possibilities, you have done your part, epistemically speaking; now it is up to the world to be obliging (or not). Epistemic anxiety is valuable from the perspective of anti-risk epistemology because it motivates one to take the steps that, when the world is obliging, are the steps required to rule out the error-possibilities that prevent one from being in a position to know. By ruling out those possibilities, again if the world is obliging, one puts oneself in a position to know the relevant proposition.

## Other accounts of epistemic anxiety

In this section, I compare my account of epistemic anxiety to two extant accounts in the literature: Jennifer Nagel’s (2010a) and Juliette Vazard’s (2018, 2021). I argue that my account has an explanatory advantage over Nagel’s, because it explains how epistemic anxiety is able to do the epistemological work to which Nagel puts it, and that it is preferable to Vazard’s account for two reasons: my account, but not Vazard’s, distinguishes epistemic anxiety from anxiety more broadly; and my account captures all instances of emotional episodes of epistemic anxiety, while Vazard’s cannot get a grip in cases where nothing is at stake for the subject. I then argue that Vazard’s overall picture of the relationships between epistemic anxiety, doubt and inquiry is problematic, and argue that a better picture is one suggested by Hookway (1998), on which doubt is identified as epistemic anxiety, and doubt motivates inquiry.

Jennifer Nagel offers an influential account of epistemic anxiety as a “force” (2010a, p. 408) that motivates subjects to gather evidence and reason carefully in certain contexts, such as those in which the practical costs of false belief would be very high. She appeals to epistemic anxiety to undermine the motivation for stakes-sensitive theories of knowledge, on which what is at stake for a subject can make a difference to what she can know. Nagel proposes a view she calls *adaptive invariantism*, according to which the standards for knowledge are invariant, and our reluctance to ascribe knowledge to subjects in high-stakes contexts arises from “an invariant expectation that subjects will think adaptively” (409): that they will invest more cognitive resources into making judgements when there is greater anticipated cost in inaccuracy, or greater anticipated reward in accuracy. Nagel uses ‘epistemic anxiety’ to name the “heightened need for greater evidence and more thorough processing that is characteristic of high-stakes situations” (414). However she does not have much to say about the nature of epistemic anxiety, and in particular, does not conceive of it as an emotion, as Vazard notes (2021, p. 6922).

Because of this, Nagel’s account of epistemic anxiety does not explain *how* epistemic anxiety can do the epistemological work to which she puts it. My picture, on which epistemic anxiety is an emotional response to epistemic risk, can. In certain contexts, for example high-stakes contexts, uneliminated error-possibilities are salient; in high-stakes contexts, salient because their obtaining would be practically costly for the subject.^13^ One’s epistemic anxiety represents the relevant error-possibility to one as robustly possible and as negative; that is, as a risk-possibility, with the relevant risk event being one’s forming a false belief. One’s epistemic anxiety is experienced as aversive. These representational and affective aspects of one’s emotional experience combine to generate motivational power: one is immediately motivated to engage in behaviours aimed at reducing the relevant risk, that of forming a false belief. Such behaviours include just the behaviours Nagel cites: evidence gathering and more careful reasoning. My account of epistemic anxiety as an emotional response to epistemic risk can thus be understood as an elaboration of Nagel’s account, which explains how epistemic anxiety is able to function in the way that Nagel posits.

Vazard too offers an account of epistemic anxiety as an emotion, which she likewise takes to be an elaboration of Nagel’s account. However for Vazard, epistemic anxiety is not an emotional response to epistemic risk. Rather, it is an emotional response to practical risk: risk regarding one’s practical interests. Vazard posits epistemic anxiety as the emotion that gives motivational power to what she, inspired by C. S. Peirce, calls “real doubt”, a kind of doubt that is “motivated by practical interests and which acts as a reason for mental and physical action” (2021, p. 6922). To illuminate the notion of real doubt, Vazard gives an example. Suppose that I doubt that it will rain tomorrow. As I am a philosopher, whether it will rain tomorrow has very little, if any, bearing on my practical stakes. Thus, Vazard holds, my doubting that it will rain tomorrow is not a “source of preoccupation” for me; it is not “accompanied by any specific phenomenology and it won’t motivate me to launch any specific action plan” (6919). My doubt is thus not a *real* doubt. Compare a farmer who doubts that it will rain tomorrow, while her crops are threatened by drought. She would be “much more likely to experience negative feelings with respect to the situation, to see it as a problem which needs to be solved, and to be moved to action as a result” (6919). The farmer’s doubt is thus a real doubt.

For Vazard, epistemic anxiety plays a role in generating real doubt, but cannot do this by itself. Rather, epistemic anxiety generates real doubt only in combination with another epistemic emotion: the feeling of uncertainty. This is a “metacognitive experience … aimed at monitoring the safety of a belief by tracking the fact that the method used to reach that belief produces true belief also in nearby possible worlds” (6930). The feeling of uncertainty is triggered when one’s belief is unsafe: when one fails to form a true belief in close possible worlds in which one forms a belief via the same method. Real doubt is generated, on Vazard’s account, “when epistemic anxiety appraises the matter as implying a possible threat, and feelings of uncertainty signal that a belief is unreliable” (6931). In such a situation, subjects are immediately motivated to deploy the “costly cognitive strategies constitutive of doubt – deliberation, reasoning, etc. about whether *p*” (6931). Real doubt is thus a “two-step model involving the intervention of two affective states: (1) an emotional episode of epistemic anxiety signaling that the proposition involved implies a possible threat, or possible negative outcomes and (2) a feeling of uncertainty signaling the lack of epistemic safety of a belief in a proposition” (6931).

Vazard argues that real doubt is so cognitively costly that it is adaptive for us to experience it only in the face of high practical stakes (6921). Thus it is crucial that the threats that trigger epistemic anxiety are not themselves epistemic, but are threats to one’s practical interests. In the rain example, it is the practical threat of drought that triggers the farmer’s epistemic anxiety, and thus makes her doubt ‘real’ where mine is only ‘paper’. Then Vazard’s epistemic anxiety does *not* have epistemic risk as its formal object. Vazard does not, in fact, have much to say about what is the formal object of epistemic anxiety. Following Kurth (2015, p. 5), she holds that the formal object of anxiety in general is “problematic uncertainty”, which in turn she defines as “potential negative outcomes (implied by some particular event or situation) over which we lack information” (6922). This is similar to how I understand risk: one faces a risk where one faces the robust possibility of a negative event obtaining. However Vazard explicitly adds that one must lack information about whether the possibility will obtain. We can thus say that, for Vazard, the formal object of anxiety is risk *plus* uncertainty.

But Vazard does not specify a distinct formal object for epistemic anxiety. She gives some examples of possible material objects of epistemic anxiety, writing that instances of epistemic anxiety “have as object a certain state of affairs which can be expressed by a proposition such as ‘the bank will not be open on Saturday morning’ or ‘the train does not stop at Foxboro’, where this possibility is evaluated as implying a possible threat” (6922). A ‘possible threat’ is defined as “potential practical costs” (6923). But note that the risk-possibilities denoted in these propositions are not epistemic risk-possibilities: they are possibilities in which, for example, *the bank is not open on Saturday morning*, rather than in which *S has a false belief that the bank is open on Saturday morning*. In the rain example, the farmer’s epistemic anxiety is about the practical risk event of drought; this is its material object. Then for Vazard, the material objects of episodes of epistemic anxiety are bog-standard risk events, not epistemic risk events. This suggests that, for Vazard, the formal object of epistemic anxiety is just the same as the formal object of anxiety: risk plus uncertainty.

If this is so, then it is unclear in what sense Vazard’s epistemic anxiety is a distinctly epistemic form of anxiety. The anxiety the farmer feels when her crops are threatened by drought is simply anxiety about some non-epistemic event obtaining. Though this anxiety has an epistemic element, this is only the epistemic element shared by all emotional episodes of anxiety, on Vazard’s account: one lacks information about whether the risk-possibility will obtain, thus it is represented as uncertain. And note too that the kinds of behaviours that the farmer’s anxiety would motivate are not the kinds of epistemic risk-reduction behaviours that epistemic anxiety is supposed to motivate: the farmer would be motivated to, for example, gather water so that she can water her plants, or invest in a better irrigation system. It is not plausible that she would be more motivated than I would to engage in *epistemic* risk-reduction behaviours, aimed at reducing the risk of having a false belief about whether it will rain tomorrow: in particular, I hold that I, just as the farmer, would suspend judgement about whether it will rain tomorrow until I had gathered more evidence, for example by checking the weather forecast. It is therefore unclear what work epistemic anxiety, on Vazard’s understanding, could do that anxiety couldn’t do just as well. This provides us with one reason for preferring my account of epistemic anxiety to Vazard’s: Vazard’s account is insufficiently specific to distinguish it from anxiety in general, thus to show what is distinctly valuable about epistemic anxiety.

Vazard’s picture of epistemic anxiety is in another way insufficiently general. As epistemic anxiety is, for Vazard, an emotional response to threats to one’s practical interest, it cannot get an explanatory grip in cases where one’s practical interests are not threatened. But there are cases in which it is plausible that a subject has the same kind of emotional experience, and is motivated to engage in the same kind of epistemic risk-reduction behaviours, as in cases of ‘real doubt’, but where there is no threat to her practical interests. Nagel discusses a case like this, in which a subject is considering the possibility that what looks like a red table to her may in fact be a white table illuminated by red trick lighting. Nagel writes that this subject “would glance up to check [the lighting] prior to making a judgement about the colour of the table” (2010b, p. 303).^14^ Here, the same behaviour is triggered as in a high-stakes case: the subject feels the need to gather more evidence before forming her belief. But in this case, there isn’t anything practically at stake for the subject regarding the colour of the table. She is motivated to check the lighting simply because considering the possibility that it is illuminated by red trick lighting has “supplied [her] with some concerns about error” (301). Vazard cannot allow that these concerns are the manifestation of epistemic anxiety. This is a shame, as the emotional experience the subject has in this case seems the same in all important respects as does Vazard’s subject undergoing ‘real doubt’: both subjects feel the need to, and consequently are motivated to, gather more evidence before forming a belief.

My account of epistemic anxiety does better than Vazard’s on both counts. It is specific enough to distinguish epistemic anxiety from anxiety in general. On my account, epistemic anxiety is a subspecies of anxiety whose formal object is epistemic risk, the risk of forming or holding a false belief. (The material object of an emotional episode of epistemic anxiety will be a particular event of forming or holding a false belief.) And it is general enough to allow that subjects can experience epistemic anxiety in the absence of practical risk. For epistemic anxiety is triggered in the presence of epistemic risk, which needn’t correspond to risk of other kinds. Indeed, in some cases, an epistemic risk event’s obtaining would be practically beneficial for one. Consider for example my belief that my ticket in a fair, ten million ticket lottery is a loser: my epistemic anxiety prevents me from forming a belief in this proposition, though of course it would be practically very good for me if this belief turned out to be false.

Vazard’s overall picture of epistemic anxiety and its relationship to doubt is also problematic. Recall that for Vazard, real doubt is generated by the combination of two affective states: epistemic anxiety and the feeling of uncertainty. She writes that “these combined affective states mediate the deployment of costly cognitive strategies *constitutive of doubt* – deliberation, reasoning, etc. about whether *p*” (2021, p. 6931, my emphasis). Here Vazard seems to identify doubt with epistemic behaviours aimed at reducing epistemic risk: deliberation, reasoning, and so on. This entails that someone who does not undertake these behaviours does not have a (real) doubt. But this is implausible. Someone who feels uneasy about the possibility of a belief she has, or is about to form, being false might not engage in these epistemic risk-reduction behaviours for a number of reasons. She might not have the time to engage in further deliberation before she must make a decision,^15^ or she might drop down dead from a heart attack before she is able to reason further.

I suggest that it is more plausible to hold that doubt comes before epistemic risk-reduction behaviour, and motivates this behaviour. Then a better picture of the relationship between doubt and anxiety is one on which doubt is *identified* with epistemic anxiety: doubt is the epistemic emotion that draws a subject’s attention to an epistemic risk-possibility, and motivates her to reduce epistemic risk. The epistemic risk-reduction behaviours in which she consequently engages – evidence-gathering, careful reasoning, and so on – can then be thought of as constitutive of *inquiry*. Two advantages to this conception of the relationship between epistemic anxiety and doubt immediately present themselves. First, it shows that what might seem an exotic theoretical notion, that of epistemic anxiety, actually has common currency in everyday life under the name of ‘doubt’. Second, this picture of doubt is more ontologically parsimonious than is Vazard’s: to endorse my account, one only need countenance one epistemic emotion, rather than two distinct epistemic emotions working in tandem to create some new state.

This picture comes close to the Peirce-inspired account of doubt developed by Christopher Hookway, from whom Nagel takes the terminology of “epistemic anxiety” (see Hookway 1998, p. 222). For Peirce, doubt is “an uneasy and dissatisfied state from which we struggle to free ourselves” (1986, p. 38). This aversive aspect of doubt gives it motivational power, and what it motivates is the activity of inquiry: “The irritation of doubt causes a struggle to attain a state of belief. I shall term this struggle *Inquiry*” (38). Hookway interprets Peirce’s doubt as a kind of anxiety, writing that “when I come to doubt a proposition, I become *anxious* about any tendency to belief it that I still possess; I shall also become anxious about any other beliefs I hold which may depend on it” (1998, p. 218). Doubt, On Hookway’s account, is then conceived of as “anxiety about any inquiry that relies on a doubted proposition”, and this anxiety “motivates us to remove the doubt that attaches to the proposition” (221). My picture of epistemic anxiety as an emotional response to epistemic risk could be used to flesh out Hookway’s Peircean picture of doubt, though this would be a task for future work.^16^

## Conclusions

To sum up, I have in this paper offered an account of epistemic anxiety as an emotional response to risk. I argued that epistemic anxiety, like anxiety in general, is generated in response to two kinds of (epistemic) risk: normic and probabilistic. However I showed that epistemic anxiety is nevertheless still valuable from the perspective of anti-risk epistemology, which must be understood as appealing to modal risk. This is because epistemic anxiety motivates subjects to reduce normic epistemic risk, and reducing normic epistemic risk and reducing modal epistemic risk are normatively coincident goals: one cannot aim for one without aiming for the other. I argued that my account of epistemic anxiety has advantages over extant accounts. It is more fleshed out than Nagel’s, who says of the nature of epistemic anxiety only that it is a ‘force’ with a particular motivational power. My picture of epistemic anxiety as an emotion explains how epistemic anxiety has the motivational power that Nagel attributes to it. Thus my account has an explanatory advantage over Nagel’s, though I hold that nothing in my account is incompatible with what Nagel says about epistemic anxiety. In contrast, my account conflicts with Vazard’s picture of epistemic anxiety as an emotional response to potential practical threats in one’s environment. But my account is preferable to Vazard’s, as it avoids two problems facing her account: that it cannot distinguish epistemic anxiety from anxiety in general, and that it cannot capture all instances of epistemic anxiety. I then compared my account of epistemic anxiety to Hookway’s Peircean picture of doubt, and noted interesting similarities between the two. A worthwhile future research project, I suggest, would explore the relationships between epistemic anxiety, doubt and inquiry, in dialogue with Hookway and Peirce.

## Footnotes

1 I have presented earlier versions of this paper at the Luck, Risk and Competence workshop at the University of Seville and for the Varieties of Risk AHRC-sponsored joint research project at the Universities of Edinburgh and Stirling, and I am grateful to all those who gave me feedback on those occasions. Special thanks are due to Matt Jope, Angie O’Sullivan and Martin Smith for in-depth and stimulating discussions that have shaped my thinking on these topics. I am also very grateful for the thoughtful and patient feedback of the reviewers at Synthese, which significantly improved this paper.

2 ‘Threat’ and ‘risk’ in the sense I am interested in are synonymous. Compare these definitions of ‘risk’ to the Cambridge English Dictionary (2013) definition of ‘threat’ as “unwanted possibility … the possibility that something unwanted will happen”. Further, the Merriam-Webster dictionary (2020), the Collins thesaurus (2013) and the Macmillan thesaurus (2018) all list ‘risk’ and ‘threat’ as synonyms. Henceforth, I will primarily use ‘risk’ to refer to the phenomenon under consideration.

3 Some philosophers reject characterisations of epistemic possibility in terms of what some subject or group of subjects know. For example, Dougherty and Rysiew 2009, argue that P is epistemically possible for a subject S only if P is compatible with S’s evidence, which is *not* identical to what S knows: “what is epistemically possible for a subject is those things which his *evidence*, rather than what he *knows*, does not rule out” (2009, p. 127). However it is standard to define epistemic possibility in terms of what is known by a subject or group of subjects, as I have done here; see Hacking (1967, p. 149), DeRose (1991, pp. 593-4), Anderson (2014, p. 597). Delineating the relevant group of subjects for a given statement of epistemic possibility is a vexed matter, but delving into this debate would take us too far afield from the arguments of this paper.

4 I do not mean to suggest that agents are never motivated to engage in risk-reduction behaviours unless they experience anxiety. Rather, the value of anxiety lies in its efficiently directing its experiencer’s attention to a risk-possibility, and immediately motivating her to take steps to reduce the relevant risk – that is, it motivates her to takes steps to reduce the relevant risk without requiring that she has an independent desire to reduce this risk.

5 Ebert, Smith and Durbach dispute the probabilities Pritchard offers for Case 2 as “unreasonably high” (2020, p. 436). In support of this, they note that they were offered odds of 5000 to one on a bet similar to Pritchard’s first condition from a bookmaker, and were denied quotes on the second and third conditions due to their “extremely improbable nature” (437, n. 8). Supposing that each of the three conditions has a probability of 5000 to one, and treating the conditions as mutually independent, Ebert, Smith and Durbach derive a probability of 1 in 125 billion for the bomb going off in Case 2. With these more realistic probabilities in place, the probabilistic account straightforwardly predicts that the bomb going off in Case 1, with odds of 14 million to one, is much riskier than its going off in Case 2, with odds of 125 billion to one. Pritchard pre-empts this objection, arguing that “even though one can always dispute the assignment of probabilities in a particular case, it ought to be clear that there will inevitably be pairs of cases of this general type” (2015, p. 443) – that is, cases where the probability of two risk events is the same, but their modal closeness differs – so “even if they manage to make this claim stick in this particular pair of cases, it ought to be clear that the underlying problem is not thereby solved” (444).

6 This is a metaphysical condition: to ‘keep initial conditions fixed’ means that the relevant possible worlds are identical to the actual world in certain respects up until E’s obtaining (see Lewis 1973, pp. 566-7).

7 Ebert, Smith and Durbach’s primary objections to the modal account centre on its having the consequence that any actually obtaining risk event is maximally risky, and the corollary that we cannot calculate the risk of an event without taking a stand on whether it will actually obtain. This leaves the modal account of risk ill-suited to play important roles we expect of a notion of risk (2020, pp. 441-2).

8 As Ebert, Smith and Durbach note (2020, p. 442), Pritchard must simply assume that the detonation-triggering conditions do not obtain in Case 2, for if they all did obtain, the world in which the bomb goes off would be the actual world, and so maximally close.

9 Could the modal account of risk be relativised to a body of evidence? Not really. A body of evidence can suggest that a world is close, but it cannot make this the case. Closeness orderings are about the extent to which worlds resemble each other, not the extent to which any particular body of evidence (again, perhaps except total evidence) suggests that worlds resemble each other. Further, given the epistemological work that Pritchard puts his modal account of risk to, it would not be desirable for him to develop a picture of risk that is like the modal account, but relativised to evidence (on which, for example, the risk of an event E is a matter of how close is the E-world that one’s body of evidence suggests is closest to the actual world). I return to this point in § 4.

10 It doesn’t matter why my ordinarily trustworthy friend has come out with false testimony on this occasion. Perhaps a brain lesion has formed overnight and caused her to have these odd beliefs (cf. Plantinga, 1993).

11 Here, ‘the belief one would thereby come to have’ should be read *de dicto*, not *de re*. Then beliefs in necessary truths can be risky, if formed in a way such that one was likely to form a false belief in some other proposition.

12 One can aim to bring about a situation in which *someone else* has a justified belief but does not know that P, for example by planting misleading evidence for them to find. Smith considers the “somewhat contrived” case in which one is about to have one’s memory wiped, and plants misleading evidence for one’s future self to find (2016, p. 16). He argues that cases like this should be thought of as cases where one attempts to manipulate the beliefs of another: one’s future self is treated as a distinct epistemic subject.

13 As I will argue later in this section, high-stakes contexts are not the only contexts in which epistemic anxiety can be generated by the salience of uneliminated error-possibilities.

14 See also Hawthorne’s 2004 discussion of “anxiety-provoking inferences”, where S knows that P and tries to infer from P that some far-fetched error-possibility does not obtain, but instead of coming to know that this possibility does not obtain, S loses her belief that P (2004, pp. 160-1). The error-possibilities needn’t be such that S would be practically worse-off if the possibility obtained. They are simply possibilities in which S holds a false belief. Note that Nagel cites Hawthorne as giving anxiety a similar role in his epistemology as she does (2010a, p. 429, n. 2).

15 It is important to Nagel that someone who experiences epistemic anxiety can weigh up the importance of satisfying epistemic anxiety by gathering evidence, reasoning carefully and so on, against other concerns, for example the importance of coming to a decision quickly (2010a, p. 415).

16 A reviewer at Synthese raised the following problem for my identifying Peircean doubt with epistemic anxiety: suspending judgement is a perfectly legitimate way of reducing epistemic risk, and indeed sometimes is a subject’s only way of reducing epistemic risk (for example, in lottery cases), so ought to quell epistemic anxiety; but Peircean doubt is satisfied only by the subject’s forming a belief, so ought to be present (and irritating) for as long as the subject remains in suspension. I am inclined to think that this problem is on Peirce’s end: if a subject realises she is not in a position to properly settle a question (for example, the question whether her lottery ticket is a loser), then she ought not to inquire; insofar as Peircean doubt motivates subjects to inquire in these situations, it is disvaluable. It would be more adaptive if doubt did not motivate subjects to inquire in such cases. Thus it would be more adaptive if doubt could be quelled, at least sometimes, by suspending judgement. This is reason, I hold, to modify Peirce’s account of doubt in the direction of my account of epistemic anxiety. But again, this is a task for future work.
